# Climatic shocks associate with innovation in science and technology

**DOI:** 10.1371/journal.pone.0190122

**Published:** 2018-01-24

**Authors:** Carsten K. W. De Dreu, Mathijs A. van Dijk

**Affiliations:** 1 Institute of Psychology, Leiden University, Leiden, The Netherlands; 2 Center for Experimental Economics and Political Decision Making, University of Amsterdam, Amsterdam, The Netherlands; 3 Rotterdam School of Management, Erasmus University Rotterdam, Rotterdam, The Netherlands; College of Agricultural Sciences, UNITED STATES

## Abstract

Human history is shaped by landmark discoveries in science and technology. However, across both time and space the rate of innovation is erratic: Periods of relative inertia alternate with bursts of creative science and rapid cascades of technological innovations. While the origins of the rise and fall in rates of discovery and innovation remain poorly understood, they may reflect adaptive responses to exogenously emerging threats and pressures. Here we examined this possibility by fitting annual rates of scientific discovery and technological innovation to climatic variability and its associated economic pressures and resource scarcity. In time-series data from Europe (1500–1900CE), we indeed found that rates of innovation are higher during prolonged periods of cold (versus warm) surface temperature and during the presence (versus absence) of volcanic dust veils. This negative temperature–innovation link was confirmed in annual time-series for France, Germany, and the United Kingdom (1901–1965CE). Combined, across almost 500 years and over 5,000 documented innovations and discoveries, a 0.5°C increase in temperature associates with a sizable 0.30–0.60 standard deviation decrease in innovation. Results were robust to controlling for fluctuations in population size. Furthermore, and consistent with economic theory and micro-level data on group innovation, path analyses revealed that the relation between harsher climatic conditions between 1500–1900CE and more innovation is mediated by climate-induced economic pressures and resource scarcity.

## Introduction

Throughout history, humans have displayed strong capacity for creativity and innovation that have returned substantial benefits. Scientific discovery and technological innovations provided effective cure and prevention of epidemic disease, enabled increasingly efficient production of food and care for expanding populations with increasing life expectancies, and may enable societies to combat the effects of climate change on societal functioning. Given these benefits, it is not surprising that, both within and across societies, the industry as well governments place a high premium on scientific discovery and technological innovation [*1,2*]. It is therefore unfortunate that scientific discovery and technological innovation seem hard to predict and difficult to regulate. In fact, the rate of scientific discovery and technological innovations is neither linear nor progressive: It varies across cultures and fluctuates over time [*3,4*]. As much as eminent scientists are creative some but not all of the time [*5,6*], countries, cities and citizens go through periods of creative bursts and rapid cascades of technological innovations that alternate with sometimes prolonged periods of relative stability and inertia [*4,7–10*].

Time-dependent fluctuations in scientific discovery and technological innovations may fit the intuition that creative insights “come out of the blue” and that innovations are “stumbled upon.” However, an alternative and arguably more tractable perspective is that scientific discovery and technological innovations are adaptive responses to recurrent problems and imminent threats that confront individuals and their societies [*11–15*]. If true, the rise and fall of scientific discovery and technological innovation will be conditioned by exogenous pressures and threats societies and their peoples face, and which they seek to manage and avert.

The possibility that temporal fluctuations in the rate of innovations track temporal fluctuations in exogenous pressures and societal threat was examined here with annual time-series data on scientific discovery and technological innovations in Europe. We link these time-series data to the often unanticipated and sometimes rather abrupt changes in climatic conditions, and surface temperature in particular. Surface temperature can vary substantially across years due to, for example, volcanic eruptions that eject dust into the high atmosphere and reduce the amount of light reaching the Earth’s surface. It can have climatic effects that last for years, with food shortages and famine as possible consequences [*16–18*]. Surface temperature can also vary as a function of North Atlantic Oscillations in Europe and the build-up of El Nino in Latin America [*19*]. These indices and associated fluctuations in temperature also associate with impaired crop yields and food security [*20*], as well as with migration [*21*] and group conflict and interstate warfare [*22,23*].

Consistent with our main thesis, historical case studies and archaeological excavations show that, besides migration and warfare, societies can also respond to climatic shocks with ingenuity and innovation [*24,25*]. For example, in 1953 (CE; Common Era) a North Sea storm tide caused significant flooding of Northwest European coasts, leading to the loss of over 2,000 lives and extensive material damage. Affected countries responded with technological studies on the strengthening of coastal defenses and built innovative systems of dams and storm surge barriers [*26*]. Such a response echoes that of the Peruvian Chimú society (1200–1470CE), which adapted to recurrent flooding by constructing hundreds of crescent-shaped sand breaks that inhibited the intrusion of saltating sands into their irrigation canals [*27*]. Even the advent of, and subsequent innovations in agriculture in the early Holocene have been linked to rather profound changes in climatic conditions [*9,28,29*].

Although archaeological evidence and historical cases are in line with sophisticated model simulations [*4*], a systematic analysis of whether and how climate shocks affect innovation is lacking. Furthermore, the mechanisms that account for such impact remain undocumented and poorly understood. One possibility is that climatic shocks engender the social and economic pressures that, in turn, trigger scientific inquiry and technological innovation. Indeed, social and economic pressures condition creative problem solving and innovation: Studies in organizations and with R&D teams show more innovation under mild rather than no time pressure [*30*], or when organizational slack tightens [*31–34*]. And although extreme competition among firms can erode the economic rents that render innovation worthwhile, some competition incentivizes innovation [*33–35*].

Taken together, the sometimes erratic and seemingly unpredictable rise and fall of scientific discovery and technological innovation may be due to the social and economic pressures that are triggered by sharp climatic changes. We tested this possibility in one discovery study with annual time-series data for Western Europe between 1500–1900CE, and then with three confirmation studies with annual time-series data for France, Germany, and the United Kingdom between 1901–1965CE.

## Methods and results for the 1500-1900CE Time-series

**Fig 1:**
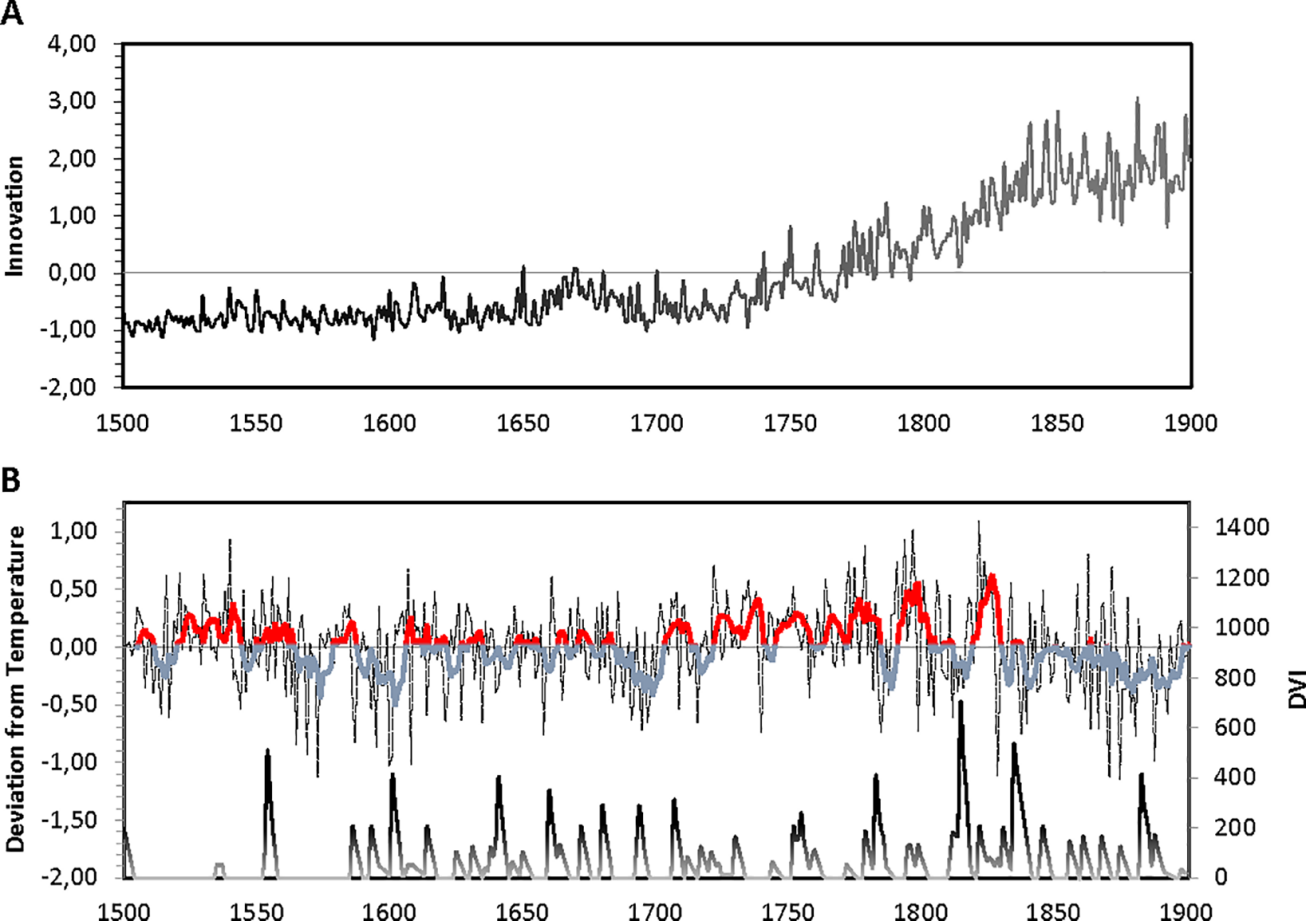
Annual time-series of innovation, temperature, and volcanic dust veils for Europe (1500–1900CE). **(A)** Innovation in science and technology expressed in factor-loading weighted average across six indicators (observed range -1.153, +3.037; *M* = 0.0, *SD =* 1.0). **(B)** Reconstructed paleo-climatic data of annual surface temperature (dotted lines) and five-year moving averages (solid lines) expressed in deviation from the period mean (8.1533°C), and Volcanic Dust Veils (observed range 0, +650; *M* = 64.214, *SD* = 99.952).

The 1500–1900CE sample provides the longest consistent and uninterrupted time-series with cross-validated and psychometrically robust annual indices of innovation [*36*], obtained by combining six historical sources on over 5,000 landmark innovations such as the development of production facilities, modes of transportation, communication technologies, and discoveries in biochemical and medical sciences (Fig 1A) [*Materials and Methods*]. Importantly, at least for this time period, reverse causality (i.e., innovations affecting climate, [*37*]) is unlikely to obscure inference. Furthermore, this sample provides a reasonable model of agrarian societies that are less technologically advanced than Western Europe nowadays and perhaps as vulnerable to climatic shocks as Western Europe between 1500–1900CE [*38*].

In the 1500–1900CE sample, innovation was related to two types of climatic shocks based on (i) the reconstructed paleo-climatic annual surface temperature for Europe [*39*], and (ii) the weighted Dust Veil Index [*16–18*], which quantifies the impact of various volcanic eruptions’ release of dust and aerosols over the years on the European continent [*Materials and Methods*] (Fig 1B). Because innovation was expected to respond to prolonged climatic shocks, we analyzed five-year moving averages in innovation as well as climatic shocks (based on both temperature and dust veils). To preclude spurious results as a consequence of analyzing non-stationary data, we detrended the annual time-series for innovation [*40*] [*Materials and Methods*].

**Fig 2:**
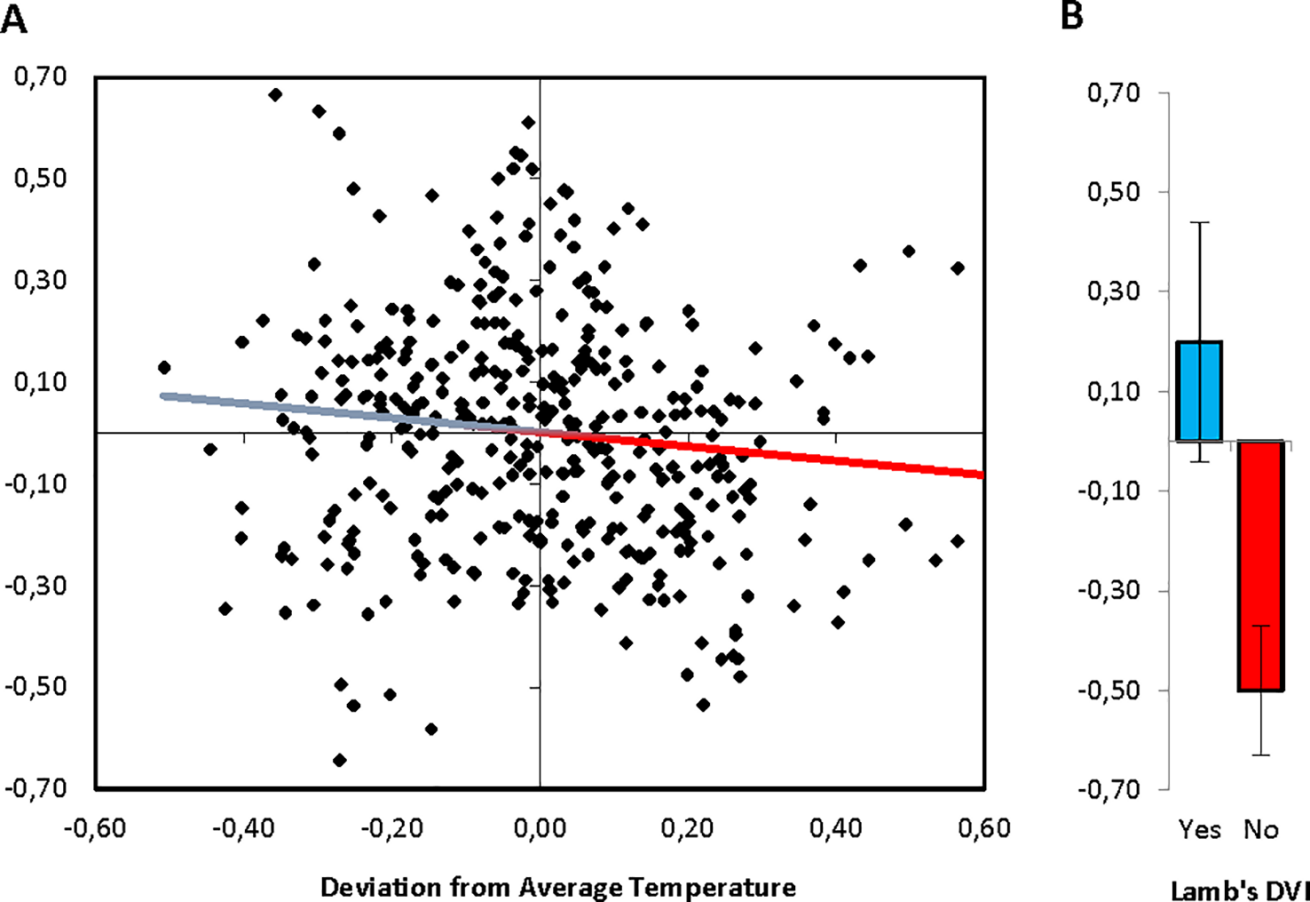
Innovation as a function of climatic shocks (Europe, 1500–1900CE). **(A)** Scatter and linear regression showing negative association between innovation and deviation from average temperature (shown are five-year moving averages, detrended series). **(B)** More innovation when volcanic dust veils are present rather than absent (five-year moving averages; shown are Mean ±1SEM).

Table 1 presents the estimation results of regressions of innovation on surface temperature and the absence or presence of volcanic dust veils. Innovation regressed on surface temperature in a negative and linear manner (standardized coefficient *b* = -0.120, *t* = -2.407, *p* = 0.017, *R*^*2*^ = 0.014; Table 1, Top Panel): thus, colder temperatures are associated with higher innovation (Fig 2A). An analogous effect was observed in a regression of innovation on a dichotomized index of volcanic dust veils (5-year moving averages; 0 = DVI absent; 1 = DVI present). The absence of dust veils significantly associated with lower innovation (*b* = -0.236, *t* = -2.309, *p* = 0.021; Table 1, Top Panel), while the presence of dust veils associated with higher innovation, albeit not significantly so at conventional significance levels (*b* = 0.084, *t* = 1.480, *p* = 0.140, total *R*^*2*^ = 0.018). A Wald test on the equality of the coefficients on DVI absent and DVI present rejected the null hypothesis that coefficients are equal with a *p*-value of 0.007. Thus, the presence of dust veils tends to be associated with significantly higher rates of innovation relative to their absence (Fig 2B). These baseline results indicate that a 0.5°C decrease in temperature (respectively, the presence of dust veils) is associated with a sizable 0.30 (0.32) standard deviation increase in innovation.

**Table 1:** Regression of innovation on surface temperature and volcanic dust veils.

**Top Panel: 1500—1900CE**				
	**Innovation**	**Innovation**	**Innovation**	**Innovation**
**Temperature**	-0.120		-0.120	
*p-value*	*0*.*017*		*0*.*017*	
**DVI absent**		-0.236		-0.241
*p-value*		*0*.*021*		*0*.*019*
**DVI present**		0.084		0.088
*p-value*		*0*.*140*		*0*.*122*
**Population change**			-0.033	-0.401
*p*-value			0.516	0.409
***Wald****(DVI absent vs*. *present)*		*0*.*007*		*0*.*005*
# observations	397	397	396	396
*R*^*2*^	0.014	0.019	0.016	0.021
**Bottom Panel: 1500—1800CE**				
	**Innovation**	**Innovation**	**Innovation**	**Innovation**
**Temperature**	-0.265		-0.276	
*p-value*	*<0*.*001*		*<0*.*001*	
**DVI absent**		-0.297		-0.294
*p-value*		*0*.*006*		*0*.*006*
**DVI present**		-0.077		-0.064
*p-value*		*0*.*264*		*0*.*348*
**Population change**			-0.282	-0.157
*p-value*			*0*.*002*	*0*.*409*
***Wald****(DVI absent vs*. *present)*		*0*.*084*		*0*.*069*
# observations	297	297	296	296
*R*^*2*^	0.070	0.010	0.100	0.035

Regressions of (detrended 5-year moving averages in) innovation on temperature and absence/presence of dust veils (Lamb’s Dust Veil Index or DVI), controlling for changes in population size, for 1500—1900CE (Top Panel) and 1500—1800CE (Bottom Panel). Coefficients are standardized. Intercepts in the regressions on temperature are suppressed to conserve space. The final three rows report the *p*-value of a Wald test on the equality of the coefficients on DVI absent and DVI present, the number of observations, and the *R*^*2*^ of the regressions.

Innovative capacity may be related to population size and changes therein, and population size may be affected by climatic shocks [*41*]. To verify that the presently observed climate—innovation linkages were robust to possible covariation in population size, we re-analyzed data with five-year moving averages in changes in population size as covariate [*Materials and Methods*]. Innovation did not relate to changes in population size (*b* = -0.033, *t* = -0.650, *p* = 0.516; Table 1, Top Panel), and the earlier observed effect of surface temperature was maintained (*b* = -0.120, *t* = -2.398, *p* = 0.017, total *R*^*2*^ = 0.016). Likewise, the effect of volcanic dust veils on innovation remained after controlling for changes in population size (*b* = -0.241, *t* = -2.349, *p* = 0.019 for DVI absent and *b* = 0.088, *t* = 1.550, *p* = 0.409 for DVI present; total *R*^*2*^ = 0.021; Wald test rejected equality of coefficients with *p* = 0.005). We conclude that the patterns shown in Fig 2A and 2B are robust to controlling for (fluctuations in) population size.

One possible concern about the analyses thus far is that, perhaps, result are obscured by the fact that (i) from 1800CE onwards relatively high levels of volcanic dust were present and (ii) innovation steeply increased (i.e., the onset of the Industrial Revolution in Western Europe) (see also Fig 1A and 1B). Put differently, even though trends are removed from all variables, it cannot be ruled out that the above results are driven by the coincidental covariation in dust veils on the one hand and the onset of the Industrial Revolution on the other. To examine this possibility, we estimated our models for the series between 1500—1800CE, thus omitting the data most strongly reflecting the Industrial Revolution.

Table 1 (Bottom Panel) gives the linear regression results (with and without controlling for population size changes). When comparing the results from the top Panel (1500—1900CE) to the bottom Panel (1500—1800CE), we can see that main results are similar in both samples: lower temperatures and the presence of dust veils are associated with higher innovation. Both the statistical significance and the magnitude of the effect of temperature on innovation are stronger in the pre-Industrial Revolution era, but the statistical significance and magnitude of the effect of dust veils are somewhat diminished relative to the longer period. All in all, we conclude that the relation between innovation and climatic shocks that this study uncovers is not driven by the Industrial Revolution.

## Methods and results for the 1901—1965CE Time-series

**Fig 3:**
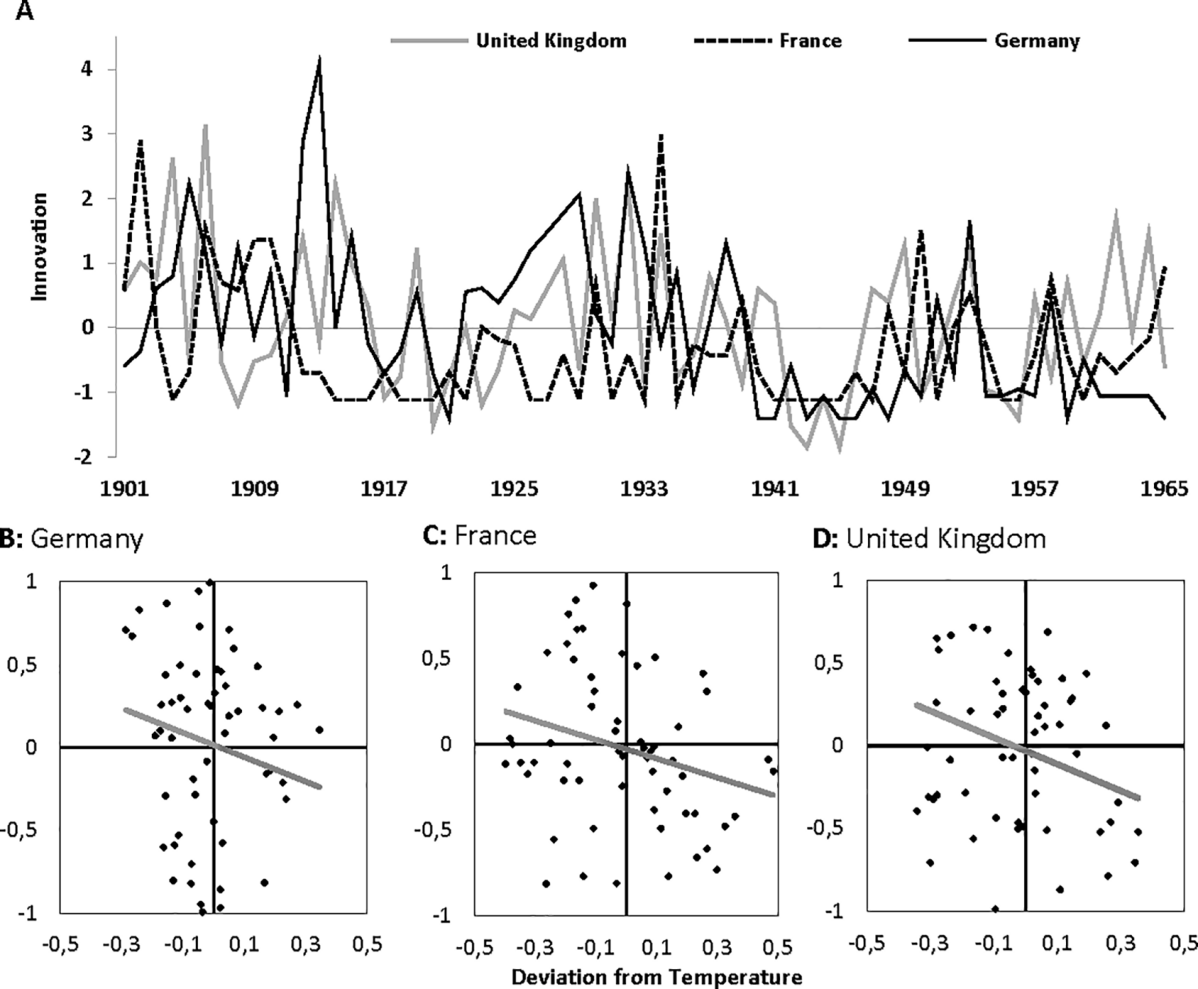
Annual time-series of innovation and temperature for France, Germany, and the United Kingdom (1901–1965CE). **(A)** Innovation in science and technology for each country expressed in factor-loading weighted averages across three indicators. **(B)** Scatter and linear regression of five-year moving averages in innovation and deviation from average temperature for Germany. **(C)** Scatter and linear regression of five-year moving averages in innovation and deviation from average temperature for France. **(D)** Scatter and linear regression of five-year moving averages in innovation and deviation from average temperature for United Kingdom.

In the period 1500–1900CE, harsher climatic conditions (prolonged cold temperatures, presence of volcanic dust veils) are thus associated with higher innovation than more benign climatic conditions. To examine the generality of this finding, we created three new annual time-series for innovation and surface temperature in France, Germany, and the U.K. between 1901–1965CE (Fig 3A) [*Materials and Methods*]. This time period begins where the discovery study ended and runs until the beginning of the Anthropocene [*37*]. While reverse causality may thus still be limited, this time period approximates contemporary conditions in technologically advanced, industrialized countries.

For the 1901–1965CE period, we were able to compute an index of innovation very similar to the one used in the discovery study, and obtained country-specific temperature data and estimates of population size [*Materials and Methods*]. As before, we computed five-year moving averages for innovation and for surface temperature, detrended the time-series, and regressed country-specific innovation on the deviation from average temperature within that country [*Materials and Methods*].

Table 2 presents the estimation results of regressions of country-level innovation in France, Germany, and the U.K. on local surface temperature. To account for multiple testing with potentially correlated independent variables and dependent variables, we first established that the multivariate effect for the linear term across all three samples was indeed significant (Hotellings *F*(9,158) = 8.778, *p*<0.001). For Germany, the linear effect was negative but not significant (Fig 3B: *b =* -0.156, *t* = -1.212, *p* = 0.230; *R*^2^ = 0.024), possibly because of the economic sanctions imposed on, and the exodus of eminent scientists and engineers from, Germany following both WW-I and WW-II [*42*]. Indeed, regressions returned significant negative linear effects of temperature on innovation for both France (Fig 3C: *b =* -0.290, *t* = -2.324, *p* = 0.024, *R*^2^ = 0.084; after controlling for changes in population size: *b* = -0.326, *t* = -2.693, *p* = 0.009, somewhat stronger) and the U.K. (Fig 3D: *b =* -0.266, *t* = -2.117, *p* = 0.039, *R*^2^ = 0.071; after controlling for changes in population size: *b* = -0.246, *t* = -1.891, *p* = 0.064, somewhat weaker). Again, the magnitudes of the observed effects are large: averaged across the three countries, a 0.5°C decrease in temperature is associated with a 0.65 standard deviation increase in innovation.

**Table 2:** Regression of Innovation on surface temperature in France, Germany, and the United Kingdom for 1901—1965CE.

	France		Germany	U.K.	
	Innovation	Innovation	Innovation	Innovation	Innovation
**Temperature**	-0.290	-0.326	-0.156	-0.266	-0.246
*p-value*	*0*.*024*	*0*.*009*	*0*.*230*	*0*.*039*	*0*.*064*
**Population change**		0.318			-0.179
*p-value*		*0*.*012*			*0*.*175*
# observations	61	60	61	61	60
*R*^*2*^	0.084	0.181	0.024	0.071	0.117

Regressions of (detrended 5-year moving averages in) innovation on temperature, controlling for changes in population size, for France, Germany, and the U.K. for 1901—1965CE. Coefficients are standardized. Intercepts in the regressions on temperature are suppressed to conserve space. The final two rows report the number of observations and the *R*^*2*^ of the regressions. For Germany, no continuous time-series of population size is available for 1901—1965CE.

## Economic pressures as mediating mechanism

The finding that harsher climatic conditions consistently associate with more innovation fits studies showing that individual and group innovation benefit from some exogenous pressure [*31,32,33,43*], and that some rather than no inter-firm competition and resource scarcity can incentivize innovation [*34,35*]. We hypothesized that this climate–innovation link may be partly due to climate-induced economic pressures and resource scarcity. For the 1500–1900CE series, we were able to examine this possibility by including annual time-series data on wheat prices [*Materials and Methods*]. Wheat crops are sensitive to climatic conditions [*20, 24*] and because wheat formed a major part of the diet in large parts of Europe over this period [*44*], wheat prices provide a reasonable basic proxy for economic scarcity. As before, we used five-year moving averages and detrended the time-series.

**Fig 4:**
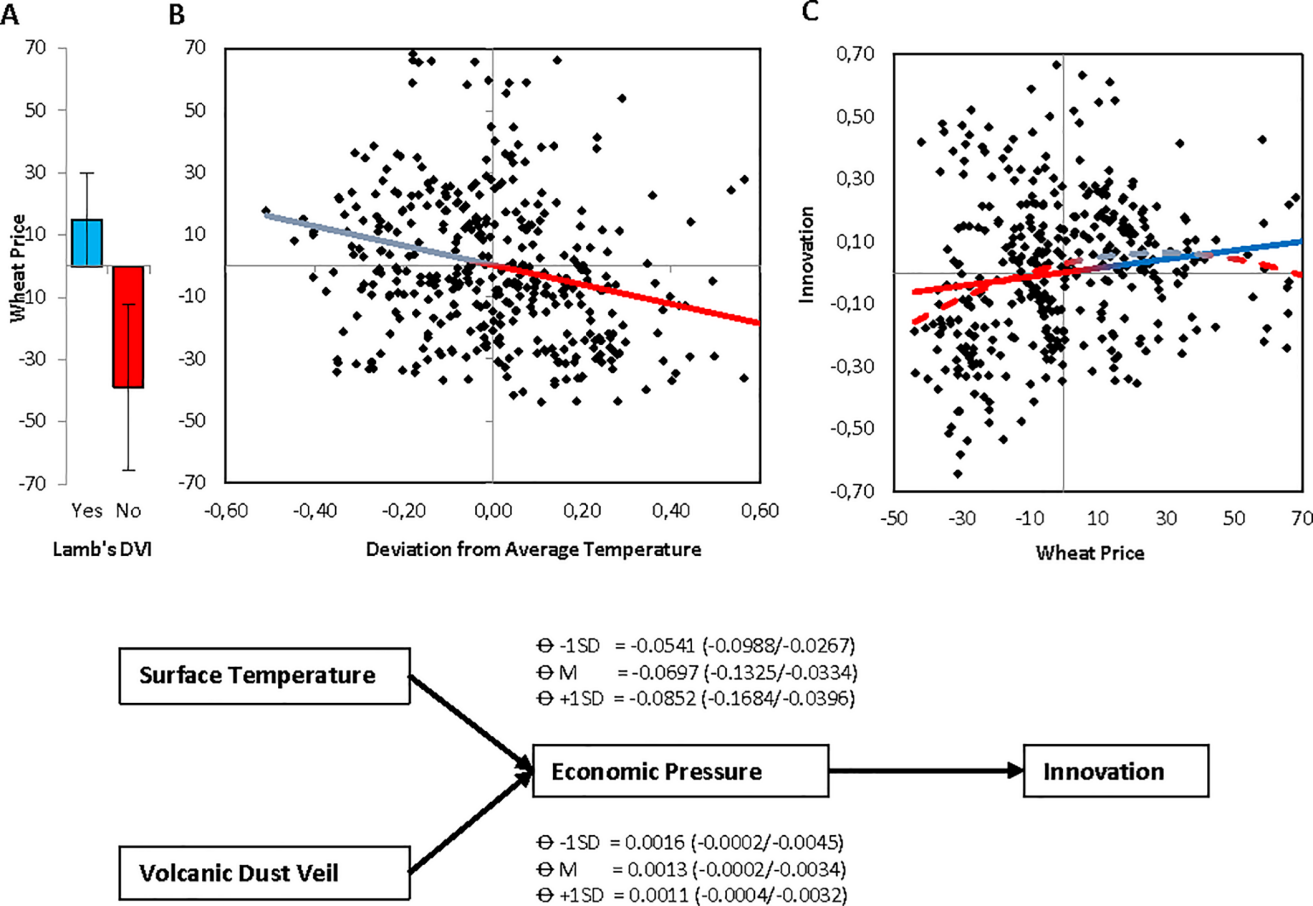
Economic pressure mediates climatic conditioning of innovation (Europe, 1500–1900CE). **(A)** Wheat price is higher when volcanic dust veils are present rather than absent (five-year moving averages; shown are Mean ±1SEM). **(B)** Scatter and linear (solid) and quadratic (dotted) regression lines for the association between innovation and wheat price (shown are five-year moving averages, detrended series). (**C)** Indirect path model showing five-year moving averages in climatic shocks (prolonged deviation from average temperature; presence of volcanic dust veils) impact on innovation through wheat prices. Shown estimates based on MEDCURVE using 5,000 bootstraps and 95% Confidence Intervals. For the temperature–wheat–innovation (dust veil—wheat—innovation) path, instantaneous indirect effects θ are shown at Mean and ± 1SD of surface temperature (dust veil) to the right (left) side of the Figure.

Table 3 presents the estimation results of regressions of wheat prices on surface temperature and the absence or presence of volcanic dust veils. We find that wheat was indeed more expensive during colder periods (*b =* -0.238, *t* = -4.860, *p*<0.001, *R*^2^ = 0.056), and when volcanic dust veils were present (*b* = -0.149, *t* = -1.453, *p* = 0.147 for DVI absent and *b* = 0.057, *t* = 0.993, *p* = 0.321 for DVI present; total *R*^*2*^ = 0.008; Wald test rejected equality of coefficients with *p* = 0.081, marginal) (Fig 4A). Both effects were robust to controlling for population size: wheat prices did relate negatively to changes in population size (*b* = -0.459, *t* = -10.620, *p*<0.001; we note the possibility of reverse causality here), but, importantly, the earlier observed effect of surface temperature was maintained when population size was controlled for (*b* = -0.233, *t* = -5.388, *p*<0.001). The effect of volcanic dust veils on wheat prices became slightly stronger after controlling for changes in population size (Wald test now rejected the equality of coefficients on DVI absent and DVI present with *p* = 0.011).

**Table 3:** Regression of wheat prices on surface temperature and volcanic dust veils.

	Wheat price	Wheat price	Wheat price	Wheat price
**Temperature**	-0.238		-0.233	
*p-value*	*<0*.*001*		*<0*.*001*	
**DVI absent**		-0.149		-0.200
*p-value*		*0*.*147*		*0*.*029*
**DVI present**		0.057		0.067
*p-value*		*0*.*321*		*0*.*191*
**Population change**			-0.459	-0.468
*p*-value			*<0*.*001*	*<0*.*001*
***Wald****(DVI absent vs*. *present)*		*0*.*081*		*0*.*011*
# observations	397	397	396	396
*R*^*2*^	0.056	0.008	0.267	0.225

Regressions of (detrended 5-year moving averages in) wheat prices on temperature and absence/presence of dust veils (Lamb’s Dust Veil Index or DVI), controlling for changes in population size, for 1500—1900CE. Coefficients are standardized. Intercepts in the regressions on temperature are suppressed to conserve space. The final three rows report the *p*-value of a Wald test on the equality of the coefficients on DVI absent and DVI present, the number of observations, and the *R*^*2*^ of the regressions.

Table 4 presents the estimation results of regressions of innovation on wheat prices. Consistent with the hypothesized effect of economic scarcity on innovation, we find that higher wheat prices were related to more innovation (*b =* 0.160, *t* = 3.224, *p* = 0.001, *R*^*2*^ = 0.026). This linear effect of wheat prices becomes stronger when we include a quadratic wheat price term as additional independent variable (*b =* 0.273, *t* = 4.743, *p*<0.001). Moreover, the significantly negative quadratic term (*b =* -0.214, *t* = -3.714, *p*<0.001, total *R*^*2*^ = 0.059) suggests that extremely high or low wheat prices undermine innovation relative to moderate price levels (Fig 4B). These effects remained strong and significant after controlling for changes in population size. We note that this inverted U-shape fits the idea that whereas some exogenous pressure benefits innovation, intense competition and economic scarcity can undermine the economic, social, and psychological resources needed to invent and innovate [*30,33,43*].

**Table 4:** Regression of innovation on wheat prices.

	Innovation	Innovation	Innovation	Innovation
**Wheat price (Linear)**	0.160	0.273	0.183	0.278
*p-value*	*0*.*001*	*<0*.*001*	*0*.*001*	*<0*.*001*
**Wheat price**^**2**^ **(Quadratic)**		-0.214		-0.213
*p-value*		*<0*.*001*		*<0*.*001*
**Population change**			0.051	0.013
*p*-value			*0*.*366*	*0*.*821*
# observations	397	397	396	396
*R*^*2*^	0.026	0.059	0.028	0.059

Regressions of (detrended 5-year moving averages in) innovation on wheat prices and squared wheat prices, controlling for changes in population size, for 1500—1900CE. Coefficients are standardized. Intercepts in the regressions are suppressed to conserve space. The final two rows report the number of observations and the *R*^*2*^ of the regressions.

We concluded analyses with computing indirect path estimates for the linear impact of climatic shocks on innovation through the curvilinear impact of economic scarcity on innovation [*Materials and Methods*] [*45*]. Controlling for wheat prices reduced the direct temperature-innovation linkage to non-significance (*b =* -0.061, *t* = -1.209, *p* = 0.227), and the indirect climate–pressure–innovation path (instantaneous indirect effect) [*45*] was significant (Fig 4C). Although the direct dust veil–innovation linkage remained significant after controlling for wheat prices (*b =* 0.011, *t* = 6.916, *p* = 0.001), here also the indirect effect was significant (Fig 4C). It follows that climatic conditioning of innovation is partly predicted by climate-induced economic pressures.

## Conclusions and discussion

Creative discovery and technological innovation fluctuate across time and space. In this study, across four annual time-series that cover almost 500 years and 5,000 documented instances of scientific discovery and technological innovation in Europe, we observed that colder periods are associated with higher innovation. Furthermore, we saw that this relation between colder temperatures and higher innovation is also related to higher wheat prices, providing more direct evidence of a link with economic pressures. To some extent, at the least, fluctuations in scientific discovery and technological innovation track climatic shocks—volcanic eruptions that can suppress surface temperatures for several years, or alterations in North Atlantic Oscillations that lead to periods of relatively elevated surface temperatures and reduced economic hardship.

Innovation in the present study was broadly defined in inclusive terms. Others before us linked climatic conditions to specific innovations in, for example, agriculture [*9,28,29*] or tool use [*4,25*]. For example, one study found that prolonged changes in surface temperature in the U.S.A. not only threatened wheat production but also triggered agricultural and technological innovations that enabled wheat production in ways and areas traditionally considered unfeasible [*24*]. Such problem-driven innovation fits the adagio that “necessity is the mother of invention” and that human creativity and innovation benefit from exogenous threats and pressures [*12–14, 43*]. The broad and inclusive nature of the innovation indices studied here allows for the possibility that, in addition to innovative activity aimed at solving a specific and local climate-induced problem, human ingenuity and innovation along with tendencies for experimentation and exploration, require exogenous pressure. In fact, at the individual, group, and societal level, innovation is inherently costly and with unknown returns on investment [*46*]. It stands to reason that groups and societies engage in such costly and risky endeavors only, or especially, when there is a need to, and such felt need may be created by deteriorating climatic conditions and concomitant economic hardship.

Although data pertain to the relatively temperate climate in Western Europe and are correlational in nature, results support the general idea that climatic shocks affect innovation when and because they create socio-economic pressures to which human societies adapt. Over our period of study, we find that rising temperatures in Europe associate with lower innovation, and we attributed this inverse relation between surface temperature and innovation to climate-induced economic pressures. A possible caveat here is that our indices of innovation, both in the discovery and in the confirmation study, were derived from historical source books and events such as Nobel laureates. In both studies we used multiple sources that according to our psychometric analyses (see Materials and Methods) were sufficiently correlated to provide a single index. This generates some confidence in the validity of our data and results. In our analyses we further controlled for bias in coding (e.g., that year endings at 0, 5, 10, and 50 had relatively high numbers of innovation *[36]* [*Materials and Methods*] and we detrended time-series to reduce the possibility of statistical artefacts due to non-stationary time-series. Nevertheless, time-series of the type used here are noisy and we may have missed out important relations between climatic shocks and innovation in science and technology.

Despite measurement noise, the linear relation between climatic shocks and innovation replicated across two studies and was observed in four of the five tests. Accordingly, we suggest that new research focuses on more detailed understanding of this climate-pressure-innovation relation. In our confirmatory studies, we localized the climate—innovation relation at the country level. More fine-grained (e.g., region or city level) analyses were not feasible due to the low number of innovations we obtained from the historical sources used here. In more recent years, however, alternative indices of scientific and technological innovations are increasingly well-documented, as are climate indicators. Our current results suggests such a panel data approach *[47]* may be possible and would allow a deeper and more fine-grained understanding of the social and psychological micro-foundations of innovation triggered by climatic shocks.

Our findings reveal an association between climatic shocks and innovation but because of the nature of our data cannot establish a causal effect *[48, 49]*. At the same time, our time-series started at the end of the middle-ages and continued into the last century, a period in history where the impact of human activity on climate has been very small *[50]* so that climatic shocks can be reasonably considered as exogenous. Whereas we cannot exclude the possibility that economic activity, innovation in science and technology included, can significantly affect climatic conditions, current findings may best be understood in directional terms. Human activity in our past did not create volcanic dust veils, nor did it significantly alter surface temperature. It follows that, across the past 500 years in Europe, climate shocks may have created economic pressures and concomitant innovations in science and technology.

## Materials and methods

### Annual time-series for Europe, 1500–1900CE

The 1500–1900CE series for *innovation* is based on six different historical sources [*36*]. Sample entries from Williams [*51, 52*] include the solution for cubic equation and the invention of the watch (both around 1500CE), a manual on the irrigation of grasslands, the discovery of barium sulphite, and the binocular telescope (all around 1600CE), the discovery that hydrogen detonates when exposed to air, development of steam engines, and the publication of Newton’s optics (all around 1700CE), the discovery of infra-red solar rays and palladium, Volta’s electricity from a cell, the publication of Bell’s “system of comparative surgery” (around 1800CE), and the publication of Max Planck’s Quantum Theory, the first wireless transmission of speech, and the discovery of hormones (around 1900CE). The six indices formed a reliable composite (Cronbach’s α = 0.931), and all indices loaded on one single factor in a Principal Component Analysis (Eigenvalue λ = 4.12, *R*^2^ = 0.6738). In further analyses, we combined the six indices weighted by their factor-loadings (with *M* = 0.00, and *SD* = 1.0).

Annual surface temperature was retrieved from the European Seasonal Temperature Reconstructions [*53*]. From the data, we computed *mean annual temperature* (averaged over the four seasonal temperatures). Annual temperature associated with lower variance in temperature across the four seasons within a given year, *r*[400] = -0.509, *p*≤0.001, suggesting that hotter and colder years also had more extreme summer highs and/or winter lows than temperate years. We tracked whether variations in surface temperature were related to the North Atlantic Oscillation (NOA) index, which is the standardized (1901-1980CE) difference between the SLP average of four grid-points on a 5x5 longitude-latitude grid over the Azores and over Iceland (WDCA Paleo; [*19*]). It contains monthly (1659-2001CE) and seasonal (1500-1658CE) NOA indices, estimated using instrumental and documentary proxy independent variables from Eurasia. We computed the annual NOA mean and found in a simple regression that the five-year moving average in NAO correlated strongly and positively with the five-year moving average in surface temperature, *b* = 0.573, *t* = 13.892, *p*<0.001, *R*^2^ = 0.331.

In addition to annual surface temperature, we retrieved *Lamb’s Dust Veil Index* (DVI), a numerical index that quantifies the impact of a particular volcanic eruptions release of dust and aerosols over the years following the event in north-western Europe, from (World Data Center for Paleoclimatology Data Contribution Series #2000–075) [*54*].

For the years 1500–1868CE, we used *wheat prices* expressed in Shillings and Pence/Bushel, with one Bushel being the equivalent of 35.238 liters [*44,55*]. For the years 1869–1900CE, we computed annual averages for Europe from the monthly prices (in 1960 USD/Kilogram) from Jacks [*56*], and converted these into the metric used in Allen [*44*]. The overlap in series (1800–1868CE) provided a “test-retest” correlation of *r*(68) = 0.85, *p*<0.001, and we used the average of two indices when available.

We validated the time-series for wheat prices against the annual time-series for craftsmen’s *consumer price index* (CPI) [*44*]; the CPI is a statistical estimate constructed from the prices of a sample of representative items whose prices are collected periodically. An increase in CPI is a generally accepted measure of inflation and thus a marker of (increasing) economic pressure. Wheat price and CPI were strongly correlated indeed (*r*[400] = 0.449, *p*<0.001), and curve-fit regressions with the five-year moving average in innovation as the dependent variable and the five-year moving average in CPI as independent variable (instead of wheat price as indicator of economic scarcity) revealed a positive (non-significant) linear term (*b* = 0.058, *t* = 1.293, *p* = 0.197) and a significant negative quadratic term (*b* = -0.244, *t* = -3.532, *p*<0.001). We take this as convergent evidence for the observation that economic pressure associates with innovation in an inverted U-shaped manner.

Both wheat price and innovative capacity may be related to *population size* and changes therein, and population size may be affected by climatic shocks [*41*]. To verify that the presently observed climate—innovation linkages were robust to possible covariation in population size, we derived population size estimates from the Maddison Project (2013 version; [*44, 55*]; downloaded on March 16, 2016 from www.ggdc.net.maddison/maddison-project). The database gives a yearly estimate of population size in Western Europe from 1820–2000, and estimates at 1500CE, 1600CE, 1700CE and 1750CE. To obtain an annual estimate, missing values for the years 1501–1599CE, 1601–1699CE, 1701–1749CE, and 1751—1819CE were estimated by interpolation. This approximation ignores within century fluctuations in population size due to pandemics, famine, warfare, and other exogenous pressures [*41*], yet fits the general notion that European population size between 1500–1800CE was fairly stable [*55*]. In the main analyses, we used the five-year moving average of (detrended) changes in population size estimate as control variable.

### Time-series for Germany, France, and the United Kingdom (1901–1965CE)

For each country, we created annual time-series for *innovation* in ways similar to the one designed by Simonton [*36*] and used in the discovery study. Each series combined entries from three distinct sources. Country-based entries were derived as follows. Two historical source books [*51,57*] provided yearly entries of scientific discoveries and technological innovation, and we retained those that could be attributed to one of the three countries under study. Examples include the discovery of chlorophyll and the publication of Einstein’s theory of special relativity in Germany, Binet’s formulation of a measure of intelligence in France (both in 1905CE), the introduction in Germany of the Leica (a 35 millimeter camera with adjustable shutter), the discovery of the element rhenium in Germany, of the Auger electron in France, and the packing fraction in the U.K. (all in 1925CE), the invention of the radio interferometer that improves the resolution of radio telescopes in the U.K., the discovery of the Schwarzschild radius in Germany, and the development of homological algebra in France (all in 1955CE). In addition, we added yearly entries for the mid-career age of Nobel Laureates in chemistry, physics, and medicine obtained from [*6*]. Laureates were placed in the country where the prize-winning work was conducted.

The three series fully overlap between 1901–1965CE and for each country, and Principal Component Analysis on the three indices yielded one-factor solutions for each country (Germany: λ = 1.340, *R*^2^ = 0.457; France: λ = 1.370, *R*^2^ = 0.446; U.K.: λ = 1.268, *R*^*2*^ = 0.423). As in the discovery study, analyses were conducted on the factor-loading regression index of innovation (Germany: observed range -1.11, +3.917, *M* = 0.00, *SD* = 1.0; France: observed range -1.849, +3.133, *M* = 0.00, *SD* = 1.0; U.K.: observed range -1.407, +4.079, *M* = 0.00, *SD* = 1.0).

Annual surface temperature for each country was obtained from (IGBP PAGES/World Data Center for Paleoclimatology/ Data Contribution Series # 2010–047) [*17*]. The dataset provides Gridded April-September multiproxy European temperature reconstructions, expressed in °C anomalies relative to the 1961–1990CE average. We took the surface temperature 5°x5° grids corresponding to the hemispheric coordinates for Berlin (German-series), Paris (France series), and London (U.K. series). Annual estimates of country’s population size were obtained from the Cross-country Historical Adoption of Technology (CHAT) dataset [*58*]. For the German series, not enough entries were available (due to missing observations for the WWI and WWII periods) to perform meaningful analyses. For the U.K. and France series, we used five-year moving averages of the (detrended) change in population size in the robustness analyses.

### Data preparation and analytic strategy

As we expect innovation to be related to prolonged climatic shocks, we analyzed five-year moving averages in innovation as well as climatic shocks (based on both temperature and dust veils) and did the same for population size and wheat prices. An important concern is the use of non-stationary time-series in the regressions, since they can lead to spurious results [*40*, *59*], we detrended the annual time-series for innovation, population size and wheat prices the annual series and for the series based on five-years moving averages. Furthermore, Simonton [*36*] noted that innovations in science and technology more often appeared in years ending at 0, 5, 50, or 00, and attributed this to a dating bias among historians documenting innovations. To avoid inaccurate conclusions regarding cyclical tendencies in innovation, we thus controlled for four dummy variables (one for each of these year-endings, coded as 0 = absent; 1 = present). Second, and in addition to the four dating-bias dummies, we partialled out the linear and quadratic trends found in the data, and used the Augmented Dickey–Fuller test (ADF) to test the null hypothesis of whether a unit root was present in the detrended time-series. In all analyses, involving annual and/or five-year averages, the null hypothesis was rejected [*60*].

We used standard multiple regression models to estimate innovation and wheat price as a function of surface temperature (or the absence/presence of volcanic dust veils), and with and without controlling for changes in population size. All models included an intercept (except when dummy variables for both the absence and the presence of volcanic dust veils were included), and were interpreted in terms of the explained variance *R*^*2*^ and the significance of standardized regression coefficients (see Tables 1 through 4). Indirect paths (shown in Fig 4D) were estimated with MEDCURVE (IBM SPSS v23) [*45*]. We specified the X → M and X → Y paths as linear and the X → Y path as quadratic, and used 5000 bootstraps to estimate the 95% Confidence Intervals for the test parameter θ (Theta). The instantaneous indirect effects *θ* are provided at the sample mean of X and at ±1SD above/below the sample mean. Significant *θ* (i.e., the 95%CI does not include zero) indicates the presence of an indirect path (*X* → *M* → *Y*) [*45*].

Our use of five-year moving averages may result in tighter confidence for the residual variance than is present in the actual data, and thus may lead to an overestimation of the effects. We verified, first, whether results replicate when using the (detrended) annual series rather than five-year moving averages. This was the case for all relations reported in the Main Text, except for the direct association between annual temperature and innovation. Second, we performed our analyses on sequential five-year periods, taking the average within each period and thus having 1/5^th^ of the number of observations but also no built-in autocorrelation. Again, we replicated our results and were able to draw the same conclusions (the first author can be contacted for further detail).

## Supporting information

S1 Data (XLS)

S2 Data (XLSX)
